# Expedited isolation of natural product peptidyl-tRNA hydrolase inhibitors from a Pth1 affinity column

**DOI:** 10.3934/molsci.2017.2.175

**Published:** 2017-05-12

**Authors:** Harkirat S. Sethi, Jessica L. Osier, Geordan L. Burks, Jennifer F. Lamar, Hana McFeeters, Robert L. McFeeters

**Affiliations:** Department of Chemistry, University of Alabama in Huntsville, Huntsville, AL, USA

**Keywords:** peptidyl-tRNA hydrolase, Pth1, novel target, bioactive fractionation, novel inhibitor isolation, antibiotic, antimicrobial, *Salmonella typhimurium*

## Abstract

New antibiotics and new antibiotic targets are needed to counter the development of bacterial drug resistance that threatens to return the human population to the pre-antibiotic era. Bacterial peptidyl-tRNA hydrolase (Pth1) is a promising new antibiotic target in the early stages of development. While inhibitory activity has been observed in a variety of natural products, bioactive fractionation has been a bottleneck for inhibitor isolation. To expedite the isolation of inhibitory compounds from complex mixtures, we constructed a Pth1 affinity column and used it to isolate inhibitory compounds from crude natural products. Recombinantly produced *S. typhimurium* Pth1 was covalently attached to a column matrix and the inhibitory activity isolated from ethanol extracts of *Salvinia minima*. The procedure reported here demonstrates that isolation of Pth1 inhibitory compounds from complex natural product extracts can be greatly expedited over traditional bioactive fractionation, decreasing time and expense. The approach is generally applicable to Pth1s from other bacterial species and opens an avenue to advance and accelerate inhibitor development against this promising antimicrobial target.

## 1. Introduction

Antibiotic resistance has become a significant problem in both public health and the field of medicine. As the use of antibiotics has increased, bacteria have developed tolerance or become resistant. Some bacteria, such as certain strains of *Salmonella typhimurium*, have become multidrug resistant [1,2]. As a consequence, drug treatments have become less effective and, in the United States, at least 2 million people get bacterial infections and at least 23,000 die annually [3]. Invasive Salmonella disease remains a major health problem, particularly in many parts of Asia and Africa [4]. To counter the rise of antibiotic resistance, in Salmonella spp and other pathogenic bacteria, new antibiotics and new antibiotic targets are urgently needed.

Peptidyl-tRNA hydrolase (Pth) enzymes are essential, found in all organisms, and function to recycle peptidyl-tRNAs, a critical role in protein biosynthesis [5]. Peptidyl-tRNAs are produced from stalled protein biosynthesis and expression of short ORFs or minigenes [6–8]. Buildup of excess peptidyl-tRNA is toxic to cells due to impaired translation initiation or slowed protein production as a result of tRNA starvation [6–8]. Thus, it is vital for cells to maintain Pth activity. A large majority of bacteria possess a single, highly conserved bacterial Pth (Pth1). Pth1 differs greatly from the multi-enzyme system of eukaryotes. In addition to the Pth1, eukaryotes are known to have Pth2 enzymes and a variety of Pth-domain containing proteins. Pth2s are structurally and mechanistically unrelated to Pth1s [9,10] with human Pth2 being a mitochondrial associated protein that promotes apoptosis in cells that have lost attachment to the extracellular matrix [11]. Moreover, knockout of Pth1, Pth2, or both does not impact viability in yeast [12]. Thus, while Pth activity is found in all cells, bacterial and eukaryotic systems differ significantly.

The essential function, conservation in bacterial species, and lack of an essential human homolog make Pth1 a promising target for antibiotic development. As a novel target, efficacy against drug resistant strains adds excitement to antibiotic development. Pth1 inhibition follows the proven antibiotic track of interrupting protein biosynthesis. However, since there are fewer Pth1 enzymes than ribosomes in a typical bacterial cell [7], there is a considerable stoichiometric advantage to targeting Pth1 over targeting the ribosome. Additionally, tremendous structural understanding and a growing knowledgebase of Pth1 contributes to the favorability of antibiotic development. In addition to numerous structures, understanding of complex formation [13–15]; inhibition [16,17], small molecule docking [18,19] and mechanism [20–22]. Overall, Pth1 is an outstanding new target for antibiotic development.

Herein we report the construction of a Pth1 affinity column and isolation of Pth1 inhibitory activity from a crude natural product extract in one chromatography step. Pth1 from *S. typhimurium* (StPth1) was recombinantly expressed and covalently attached to a stationary column matrix. The crude ethanol extract from *Salvinia minima* was identified to inhibit StPth1. This extract was chosen for further study due to its potent inhibitory activity and availability locally in large quantities (during the spring). Inhibitory activity from this crude natural product was isolated via two-step elution using the StPth1 affinity column. Compared to traditional bioactive fractionation, this methodology helps alleviate the current bottleneck of inhibitor isolation. Expedited Pth1 inhibitor isolation will accelerate antibiotic development against this promising new target.

## 2. Material and Methods

### 2.1. Expression and purification of StPth1

StPth1 was expressed and purified as previously reported [23]. Briefly, StPth1 cloned into the pKQV4 vector was transformed into BL21 *E. coli* cells. A single colony was used to inoculate 30 mL of LB containing 20 μg/mL of carbenicillin. The culture was grown at 37 °C overnight. This starter culture was added directly to 970 mL of LB also containing 20 μg/mL carbenicillin and grown at 37 °C until an OD_600_ of 0.6. IPTG was then added to a final concentration of 1 mM to induce protein expression. StPth1 was expressed for 3 hours at 37 °C before the culture was pelleted and stored at −80 °C. For purification, the pellet was resuspended, lysed, and applied to a metal chelation column. Eluted StPth1 was first dialyzed against a buffer of 50 mM Sodium Phosphate, 300 mM Sodium Chloride, pH 7.4, then against 20 mM Bis-Tris, 50 mM NaCl, pH 6.6, and finally concentrated via ultracentrifugation. In preparation for column attachment, concentrated StPth1 was dialyzed into 0.2 M NaHCO_3_, 0.5 M NaCl, pH 8 and further concentrated to ~20 mg/mL.

### 2.2. Pth1 enzyme activity

The enzymatic activity of StPth1 was determined as previously published [24,25] with peptidyl-tRNA produced as before [15,23]. Briefly, acid urea minigels were utilized to determine Pth1 activity level from the difference in electrophoretic mobility of heterogeneous peptidyl-tRNA (substrate) and free tRNA (product). Cleavage reaction volumes were 20 μL composed of 10 μL of reaction buffer (20 mM Tris-acetate pH 8.0, 20 mM magnesium acetate, 40 mM ammonium acetate), 0.75 μL of 120 μM StPth1, and 1.75 μL of ~1.5 μg/μL peptidyl-tRNA. The remaining 7.5 μL was either the fraction eluted from the StPth1 affinity column being tested for inhibition or a buffer. Peptidyl-tRNA was added last, after all other components for all reactions were mixed. Reactions were incubated for 30 minutes at room temperature (~22 °C) before being quenched by 8 M urea. Samples were loaded on acid-urea minigels and the products of the cleavage reaction separated at 100 V for 2 to 2.5 hours. The gels were stained overnight in methylene blue and destained for 2 hours with extensive water changes the following day. Cleavage quantitation was determined from densitometry analysis of gel images.

### 2.3. StPth1 affinity column

A 1 mL NHS-Activated Sepharose column (GE/Amersham) was utilized to crosslink with StPth1 to form the affinity column. To activate the resin, 6 mL of a 1 mM HCl solution was flowed through the column at a flow rate of 1 mL/min. Immediately afterwards, 10 mgs of StPth1 in 600 μL was applied to the column and incubated overnight at 4 °C. The next day, the column was washed with Buffer A (0.5 M ethanolamine, 0.5 M NaCl, pH 8.3) and Buffer B (0.1 M sodium acetate, 0.5 M NaCl, pH 4) described in detail below. The flow through from loading and buffer wash with measurable 280 nm absorbance (~2.5 mL) were collected to determine the amount of protein that did not remain on the column matrix. To purge any unbound StPth1 and inactivate unoccupied attachment sites, 6 mL of Buffer A was first injected followed by 6 mL of Buffer B. This was repeated twice with a 30 min incubation after the first repeated application of Buffer A. After the StPth1 affinity column was equilibrated with 2 mL of 25 mM Tris HCl, pH 7, the column was stored at 4 °C. Absorbance at 280 nm and a calculated extinction coefficient of 16,960 Abs cm^−1^ M^−1^ were used to determine the initial StPth1 concentration and that in the flow through. The amount of StPth1 attached to the column was determined by subtracting the amount of StPth1 in the flow through from the initial amount applied to the column.

### 2.4. Column chromatography for inhibitor isolation

The ethanol extract of *Salvinia minima* was resuspended in aqueous equilibrium buffer. The extract, provided by a collaborator, was prepared from dried whole plant material harvested from local North Alabama waterways that was crushed, effluxed with ethanol, and dried to a solid. Any insoluble material was removed via centrifugation at 10,000 g for 2 minutes. Crude extract, 600 μL containing approximately 100 mg of material, were applied to the previously equilibrated StPth1 affinity column and allowed to incubate for 5 minutes. The column was washed with equilibrium buffer (25 mM Tris pH 7, 5 mM NaCl) at a flow rate of 5 mL/min to remove unbound extract. At a flow rate of 3 mL/min, 7.5 mL each of boric acid elution buffer (3 mM Boric acid, pH 10) and high salt elution buffer (5 mM Tris, 2.5 M NaCl, pH 7) were applied to the column. Fractions of 0.5 mL were collected throughout the process.

### 2.5. Thin layer chromatography

TLC was run on SORBTECH 200 μm thick Silica G TLC plates with UV254. More typical solvents did not lead to migration of the unknown small molecules, however DMSO was used. For 2D TLC, 10 cm × 10 cm plates were cut from larger sheets. Approximately 2 μL of the sample to be tested were spotted onto the TLC plate using a micropipette and allowed to dry. The spot was approximately 1 cm in both dimensions from a corner of the plate. Each dimension was run to ~75% of the plate length, requiring ~20 minutes at room temperature. After the first dimension, the plate was removed and completely dried before the second dimension started. After the second dimension was run, the plate was once again removed and completely dried. Overnight incubation in an iodine chamber was necessary for visualization of the weak, non-buffer peaks.

## 3. Results and Discussion

### 3.1. Extract inhibition

**Figure 1.**
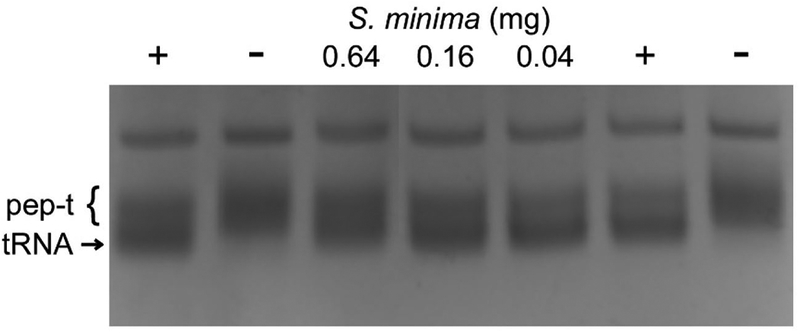
StPth1 Inhibition by *S. minima*. For positive and negative controls, the presence (+) or absence (−) of StPth1 is indicated. Increasing amounts of *S. minima* extract inhibited StPth1 cleavage of heterogeneous peptidyl-tRNA (pep-t) to free tRNA (tRNA).

Natural products from a variety of sources have been reported to have Pth1 inhibitory activity [16,26,27]. We identified a readily abundant, local natural product which demonstrated Pth1 inhibition, the ethanol extract from *Salvinia minima*. When solubilized in aqueous buffer, this extract potently inhibited StPth1, Figure 1. With the potent inhibition and local abundance, we utilized this extract to determine the effectiveness of using a Pth1 affinity column to isolate small molecule inhibitors.

### 3.2. Pth1 affinity column

To expedite isolation of the inhibitory components from complex natural product extracts, an StPth1 affinity column was generated by covalent attachment of recombinantly expressed StPth1 to the solid matrix. A 1 mL nominal volume column was utilized which had an experimentally determined void volume of ~600 μL. Thus 600 μL of 725 μM StPth1, or equivalently 10 mgs of StPth1, were loaded onto the activated column and incubated overnight at room temperature. After 18 hours, the column was flushed and StPth1 that flowed through was collected and quantified. It was determined that 6 milligrams of StPth1 was bound to the column, equating to 270 nanomoles.

To confirm the activity of covalently attached StPth1, after deactivation with Buffer A and B, the column was extensively flushed with 1 liter of equilibrium buffer. A total of 500 μL of peptidyl-tRNA (~200 μg) in the same buffer was loaded onto the column and incubated at room temperature for ~4 hours. Two milliliters of buffer was added to the column and the flow through containing uncleaved peptidyl-tRNA along with the free tRNA and peptide cleavage products were collected. The peptidyl-tRNA was completely cleaved indicating functional StPth1 was attached.

### 3.3. Isolation of Pth1 inhibitory activity

**Figure 2.**
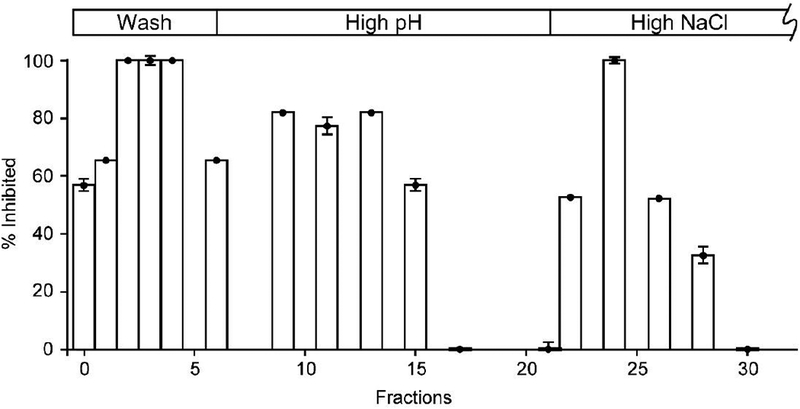
*In vitro* StPth1 Inhibition by Affinity Column Fractions. Fractions collected from the StPth1 affinity column were tested for *in vitro* inhibition of StPth1. The chromatography steps are indicated above the percent inhibition. Standard deviations from triplicate measurements were on the order of 5%, as previously reported [24], shown for those measured.

Having confirmed the activity of StPth1 on the affinity column, isolation of inhibitors from the known inhibitory extract of *Salvinia minima* was conducted. The affinity column was loaded with 100 mg of dried extract resuspended in 600 μL aqueous equilibrium buffer. After a 5 min incubation at room temperature, the column was quickly washed and inhibitor eluted with a high pH solution followed by high NaCl solution. The initial wash was limited to 3 mL to minimize inhibitor disassociation. Subsequently, 7.5 mL of both elution solutions was flowed over the column. Inhibitory activity of StPth1 was determined for each fraction, see Figure 2.

After characterizing activity, it became immediately apparent that the initial wash (5 column volumes or 5 CV) was not enough to remove excess or weakly bound inhibitors from the column. Roughly twice, or 10 CV, is estimated to return inhibition to baseline levels after addition of the crude substrate. This observation may indicate the presence of weaker binding inhibitors since the brownish color of the whole extract was not detectable past eluted fraction 4, or about 3 CV. The lack of depleted inhibitory activity also indicates that the column was overloaded with inhibitor in the extract and less initial crude extract can be used in the future.

While some degree of inhibitory activity was observed for the boric acid elution, the incomplete wash obscured the outcome. The pH 10 elution condition was based on previous studies that showed apo-StPth1 stayed in solution and was active at this high pH. Thus it was postulated that the higher pH would not be deleterious to StPth1. Regardless, more promising is the high NaCl elution. While Pth1 recognizes the peptide backbone as part of the substrate and it has been observed that hydrogen bonding appears, at least computationally [19], to contribute to small molecule binding, disrupting such interactions was a logical approach to elute inhibitors. Fortunately, the larger volume of high pH buffer returned the inhibitor activity to baseline before the NaCl elution was started. Not having the inhibitory activity in the first fraction after the buffer change (500 μL fraction, 600 μL void volume) and overall shape of the inhibition profile are positive indicators of successful chromatographic separation. Moreover, the degree of StPth1 inhibition is greatest for this elution condition suggesting effective release of inhibitors from column bound StPth1.

To validate the inhibitor isolation results, a negative control column was prepared in which there was no StPth1 covalently attached. The column was treated exactly as before, only no StPth1 was present in the aqueous buffer during overnight incubation. The *S. minima* extract was applied to this column following the same chromatography steps and inhibitory activity was determined using the same procedure as for the StPth1 affinity column. Inhibitory activity was observed in the initial flow through, trailing into the first few fractions of the boric acid elution. However, no inhibition was observed past fraction 12. This indicates that non-specific binding to the solid phase or column matrix was not responsible for the StPth1 inhibition observed, in particular not for the high NaCl elution. This also substantiated that the column needed a larger volume of initial wash buffer to return the activity to baseline before initiating elution.

To control for the potential effect of different buffers, StPth1 activity was determined in both elution buffers without any *S. minima* extract present. Neither elution buffer inhibited StPth1 cleavage of peptidyl-tRNA, alone or in combination. The boric acid buffer did have a minor effect on the activity assay, slowing migration of both peptidyl-tRNA substrate and free tRNA product. Thus boric acid fractions necessitate adjustment to neutral pH before the activity assay or could be run separately with equal volumes of boric acid added to the control reactions.

### 3.4. Post inhibitor inactivity

The activity of StPth1 bound to the column was determined after the isolation of inhibitory activity. Initially the column was flushed with 20 CV of original equilibrium buffer and peptidyl-tRNA incubated overnight as before. When no cleavage was observed, the column was further flushed with aqueous buffer for several days. However, no peptidyl-tRNA cleavage was observed. Thus it appears that the procedure leads to the inactivation of StPth1. This was unexpected since neither elution buffer condition affected *in vitro* Pth1 activity. The reason for loss of activity is not clear, though it may be possible to denature and refold StPth1 on the column to allow for re-use.

### 3.5. TLC characterization of inhibitory fractions

Samples with inhibitory activity from the Pth1 affinity column were subject to TLC. From the inability of most typical solvents to cause migration, the unknown sample was apparently highly polar, making sense for a peptidyl-tRNA mimic. However, using DMSO, migration distinguishable from the buffer only control was observed for the high salt elution fraction, but not the boric acid fraction. Thus further characterization was only possible for the high salt elution, for which two faint spots were observed with R_f_ vales of 0.12 and 0.48. Further characterization after scale-up is necessary to identify the components.

### 3.6. Estimation of time savings

The affinity column separation of StPth1 inhibitors from a complex mixture was achieved in approximately 30 minutes. However, taking into account the time to prepare the column, including StPth1 production and purification, would extend this time to 3 days in an ideal case. On the other hand, in our experience a minimum of 3 (though more typically 4 or 5) rounds of fractionation are necessary to achieve the same level of inhibitor separation. For an absolute best case theoretical scenario, each round of fractionation and activity determination could be reduced to around 2 days. More practically, each round of fractionation and bioactivity determination takes greater than a week. Therefore, given a comparison of ideals, our estimate is that column separation of inhibitor mixture is around twice as fast, more realistically 7 to 12 times faster than the traditional fractionation technique.

## 4. Conclusions

As drug discovery targeting the promising new antibiotic target Pth1 advances, the bottleneck of inhibitor isolation remains a significant barrier. Currently screening throughput is limited due to characterization of Pth1 functional characterization. Potential exists for increasing throughput, including development of higher throughput fluorescence polarization methods [28]. However, significant barriers exist for scale up of this methodology. Options like fragment based drug design are desirable, but again are limited by functional characterization.

To help circumvent the throughput limitation in functional characterization and alleviate a major limiting factor in Pth1 inhibitor discovery, we have constructed a Pth1 affinity column with the aim to expedite inhibitor isolation. We have demonstrated that inhibitory activity can be isolated from complex mixtures. While serving as proof of concept, the scale will have to be increased for more practical application. With only 270 nanomoles of Pth1 present on the column, it is not surprising that more material is necessary for characterization of the isolated inhibitory component(s). Scale up, including optimization of Pth1 covalent attachment efficiency and using amounts of solid phase column matrix, is possible and will be the immediate future priority.

While unfortunate that Pth1 activity was not restored and that the column does not appear to be reusable, the presented method does look to be a way to speed up inhibitor isolation. Furthermore, this method is likely adaptable to other Pths leading to rapid validation of the differential small molecule binding predicted from computation. Overall, this procedure should dramatically speed up inhibitor isolation, leading to identification of Pth1 inhibitors from a variety of sources.

## Abbreviations:

Pth: peptidyl-tRNA hydrolase
Pth1: bacterial Pth
tRNA: transfer RNA
IPTG: isopropyl β-D-1-thiogalactopyranoside
LB: Luria or lysogeny broth
CV: column volume
